# The combination of arbuscular mycorrhizal fungi inoculation (*Glomus versiforme*) and 28‐homobrassinolide spraying intervals improves growth by enhancing photosynthesis, nutrient absorption, and antioxidant system in cucumber (*Cucumis sativus* L.) under salinity

**DOI:** 10.1002/ece3.4112

**Published:** 2018-05-10

**Authors:** Husain Ahmad, Sikandar Hayat, Muhammad Ali, Tao Liu, Zhihui Cheng

**Affiliations:** ^1^ College of Horticulture Northwest A&F University Yangling Shaanxi China

**Keywords:** 28‐homobrassinolide, antioxidants, arbuscular mycorrhizal fungi, cucumber, NaCl stress

## Abstract

Salinity is one of the major obstacles in the agriculture industry causing huge losses in productivity. Several strategies such as plant growth regulators with arbuscular mycorrhizal fungi (AMF) have been used to decrease the negative effects of salt stress. In our experiment, 28‐homobrassinolide (HBL) with spraying intervals was combined with AMF (*Glomus versiforme*) in cucumber cultivars Jinyou 1^#^ (salt sensitive) and (Changchun mici, in short, CCMC, salt tolerant) under NaCl (100 mmol/L). Studies have documented that the foliar application of HBL and AMF colonization can enhance tolerance to plants under stress conditions. However, the mechanism of the HBL spraying intervals after 15 and 30 days in combination with AMF in cucumber under salt stress is still unknown. Our results revealed that the HBL spraying interval after 15 days in combination with AMF resulted in improved growth, photosynthesis, and decreased sodium toxicity under NaCl. Moreover, the antioxidant enzymes SOD (superoxide dismutase; EC 1.15.1.1) and POD activity (peroxidase; EC 1.11.1.7) showed a gradual increase after every 10 days, while the CAT (catalase; EC 1.11.1.6) increased after 30 days of salt treatments in both cultivars. This research suggests that the enhanced tolerance to salinity was mainly related to elevated levels of antioxidant enzymes and lower uptake of Na^+^, which lowers the risk of ion toxicity and decreases cell membrane damage.

## 1 INTRODUCTION

Salinity is among the major threats to agricultural productivity and is one of the reasons in the present time for causing huge economic losses in developing countries (Qadir et al., 2014). Therefore, the global food production must sustainably increase with every increment in population. It is a disastrous stress, and around 7% of the world land is affected by high salt levels (Hanin, Ebel, Ngom, Laplaze, & Masmoudi, 2016; Hashem et al., 2016). As sodium chloride constitutes the majority of salts in affected areas, it is considered as a significant factor in causing salinization. It causes hyperionic and hyperosmotic stress, which includes oxidative stress and nutritional imbalance (Zhu, 2002). The accumulation of sodium ion causes disruption in many cellular processes such as water conductance, photosynthesis, respiration, and plasma membrane function. It also increases electrolyte leakage (EC) and nutritional imbalance and causes toxicity in cells (Porcel, Aroca, & Ruiz‐Lozano, 2012; Volkov & Beilby, 2017). The decrease in photosynthesis causes oxidative damage by increasing the production of reactive oxygen species (ROS). The overproduction of ROS interacts with nucleic acids and lipids and causes damage to photosynthetic pigments, respiration, cell membrane structures, which eventually leads to plant demise (Mahajan, Mahajan, Tuteja, & Tuteja, 2005; Sarwat et al., 2016). Plants are equipped with many molecular and physiological mechanisms to combat with abiotic stress conditions (Ahmad et al., 2014), among which triggering of antioxidant enzymes superoxide dismutase (SOD), peroxidase (POD), catalase (CAT), ascorbate peroxidase (APX) etc. has a key role in stress tolerance (Hanin et al., 2016).

Various agronomic and physiological practices are applied to improve productivity and minimize salinity effects. However, the effective way is to introduce salt‐tolerant cultivars (Khan et al., 2006; Yildirim, Turan, & Guvenc, 2008), which have some limitations due to genetic characteristics of these crops and unfavorable environmental conditions for growth. Recently, plant growth regulators or arbuscular mycorrhizal fungi (AMF) have been given much attention which is known to be involved in regulation of growth under stress conditions (Foo, Ross, Jones, & Reid, 2013; Ruiz‐Lozano, Porcel, Azcón, & Aroca, 2012). Studies have shown that plant growth regulators are involved in regulating plant microbial symbiosis (Bucher, Hause, Krajinski, & Küster, 2014; Gutjahr, 2014; Pozo, López‐Ráez, Azcón‐Aguilar, & García‐Garrido, 2015). Brassinosteroids (BRs) are plant steroids occurring ubiquitously in the plant kingdom and are important in plant growth and development (Aghdam & Mohammadkhani, 2014). Till date, 70 forms of BRs have been isolated from plants (Bajguz & Hayat, 2009), which have a major role in differentiation and elongation of cells, tissues, protein synthesis, flowering, seed synthesis, yield, and regulation of gene expressions (Divi & Krishna, 2009; Hayat, Hayat, Irfan, & Ahmad, 2010). Studies also show that exogenously applied BRs increase protein synthesis and increase the efficiency of proton pumps (Ahmad, Azooz, & Prasad, 1979; Bajguz & Czerpak, 1996). Recently, BR role in response to various stress conditions has been studied, such as salt stress (Ahmad, Hayat, Ali, Imran Ghani, & Zhihui, 2017; Fariduddin, Mir, Yusuf, & Ahmad, 2014; Hayat, Khalique, Wani, Alyemeni, & Ahmad, 2014), cold stress (Aghdam & Mohammadkhani, 2014), heavy metal stress (Ali, Hayat, Fariduddin, & Ahmad, 2008; Hayat et al., 2014), temperature stress (Niu et al., 2016), and oxidative stress (Cao et al., 2005). Exogenously applied BRs reduces stress conditions by decreasing sodium uptake, EC, and lipid peroxidation. The foliar application of BRs under salt stress increased growth (Ahmad et al., 2017), photosynthesis, nutrient acquisition, mitotic index, and antioxidant enzymes (Ali et al., 2008; Fariduddin et al., 2014). Plants treated with BRs showed significant increment in gas exchange parameters, chlorophyll content, and leaf relative water content (Fariduddin et al., 2014).

Plants in their natural environment are colonized by a wide array of microbes, including nitrogen‐fixing bacteria, endophytes (Ilangumaran & Smith, 2017). Arbuscular mycorrhizal fungi are ubiquitous among the vast array of microbes inhabiting the rhizosphere of the plants (Egamberdieva, Wirth, Li, Abd‐Allah, & Lindström, 2017). AMF form beneficial symbiotic relations with most of the plants and improve growth by enhancing uptake of several nutrients, thus considered as an integral part of an ecosystem by existing in adverse conditions, particularly saline conditions (Hashem et al., 2016; Porcel et al., 2012). Reports have shown that despite AMF dependency on host photosynthate, they regulate efficient nutrient and water absorption by extending its hyphal network near plant rhizosphere (Berruti, Lumini, Balestrini, & Bianciotto, 2016). Reports have shown that AMF reduce salinity stress by improving host growth and hydraulic conductivity of roots and decrease lipid peroxidation (Ghazanfar et al., 2015; Hashem et al., 2016). AMF mitigate oxidative stress by elevating antioxidant system (SOD, POD, APX, CAT, etc.) of plants to overcome the increased production of ROS (Ghazanfar et al., 2015; Hashem et al., 2016; Sarwat et al., 2016; Schweiger, Baier, Persicke, & Müller, 2014). It also improves photosynthesis, chlorophyll content, and phosphorus uptake in plants (Hajiboland, Aliasgharzadeh, Laiegh, & Poschenrieder, 2010; Zuccarini & Okurowska, 2008). This study was conducted to investigate the potential role of spraying intervals of HBL and in combination with AMF in improving the physiology and lowering the toxic effects caused by salinity in cucumber plants. In this study, we focused the role of spraying intervals of HBL and in combination with AMF on antioxidant activity of cucumber plants after every 10 days and their effect on physiology and growth of cucumber plants.

## 2 MATERIALS AND METHODS

The AMF (*Glomus versiforme*) inoculum (spores and infected roots of maize*)* was obtained from the department of Horticulture, Northwest A&F University, Yangling, Shaanxi, P.R. China. The inoculum obtained was replicated for 2 years under glasshouse conditions with a day–night temperature of 32°C/27°C and relative humidity of 70%.

### 2.1 Plant materials and AMF treatments

Healthy cucumber seeds of salt‐sensitive (Jinyou 1^#^) and salt‐tolerant (Changchun mici, in short, CCMC) were surface‐sterilized with 1% sodium hypochlorite solution, repeatedly washed afterward, and swelled with distilled water overnight. Then, half of the seeds were sown in plastic trays having vermiculite and half were sowed in arbuscular mycorrhizal fungus (AMF)‐inoculated soil for better colonization with the host roots. Twenty‐five days of old uniform seedlings was transplanted to pots (30 × 30 cm) inside a plastic tunnel. The plants having AMF as a treatment received 10 g of inoculum per 0.89 kg of soil. The inoculum was placed under and adjacent to the seedling roots. The same amount of autoclaved inoculum was added to noninoculated plants and supplemented with microbial culture filtrate to provide the microbial populations accompanying the mycorrhizal fungi. There was a total of six kilograms of soil mixture in each pot, having organic matter, soil, and sand in the ratio of 1:2:1 v/v. The soil mixture properties were pH 7.45, total nitrogen (0.473 g/kg), phosphorus (0.102 g/kg), potassium (0.246 g/kg), and organic matter (9 g/kg).

### 2.2 Pot experiment and HBL treatments

HBL [28‐homobrassinolide (C_29_H_50_O_6_) molecular weight of 494.70 g/mol] stock solution of 1 mmol/L was initially prepared by dissolving HBL in a little amount of ethanol (99.9%) as a solvent. HBL concentration of 1 μmol/L concentration was prepared by dilution of stock solution, and Tween 20 (polyoxyethylene sorbitan monolaurate 0.02%) was added as a surfactant to enhance chemical absorption. Plant leaves were sprayed till saturation on both sides the day after treating them with salt stress. NaCl at 100 mmol/L (EC 10.52 ds/m) concentration was prepared and applied to plants during irrigation, and their EC values were kept constant throughout the experiment. Control plants were irrigated with tap water having an electrical conductivity of 1.19 ds/m. There were total 12 treatments T1 (control), T2 (HBL spray after 30 days), T3 (HBL spray after 15 days), T4 (AMF only), T5 (HBL spray after 30 days + AMF), T6 (HBL spray after 15 days + AMF), T7 (NaCl stress only), T8 (HBL spray after 30 days + NaCl stress), T9 (HBL spray after 15 days + NaCl stress), T10 (AMF + NaCl stress), T11 (HBL spray after 30 days + AMF + NaCl stress), and T12 (HBL spray after 15 days + AMF + NaCl stress) that were replicated three times, and 10 pots were used for each treatment.

### 2.3 Plant morphology

Plant growth parameters were determined after 65 days at the end of the experiment. Roots were uprooted, and root/shoot length was noted using a measuring tape. Shoot fresh weight and root fresh weight were noted by weighing it on weight balance. Afterward, the plant material was kept in the oven for 72 hr at 80°C (Fariduddin et al., 2014), and their dry weight was recorded.

### 2.4 Plant chlorophyll contents

Leaf chlorophyll contents were determined according to Arnon (1949). Fresh plant leaves of 0.5 g (40 days after germination) were extracted in 80% acetone (v/v). The extract was centrifuged at 10,000 × g for 10 min. The absorbance of the extract was observed on a spectrophotometer (UV‐3802, UNICO, MDN, USA) at 665‐ and 663‐nm wavelength.

### 2.5 Root activity

Root activity was determined through triphenyltetrazolium chloride (TTC) by taking 0.5 g of cucumber roots as demonstrated by Clemensson‐Lindell (1994). The absorbance of the extract obtained was measured at 485 nm by spectrophotometer (UV‐3802, UNICO, MDN, USA). The root activity was measured as per linear equation and expressed as TTC reducing intensity (mg g^−1 ^hr^−1^) from the following formula:

$$\begin{array}{cc} & \text{TTC reducing intensity}\;(\text{mg}g^{-1}{\text{hr}}^{-1})=\text{[TTC reduction mass (mg)/}\\ & \text{root weight (mg)*time(hr)]}*100 \end{array}$$

### 2.6 Electrolyte leakage

The EC from leaves was measured by taking 20 leaf disks per replication (55 and 75 days after germination) and placed in boiling tubes (with caps) having 10 ml of double deionized water(Sullivan & Ross, 1979). ECx was noted after placing leaf disks in d H_2_0. ECy was measured by heating the tubes in a water bath for half an hour at 45°C and 55°C, respectively. ECz was noted by further boiling the samples at 100°C for 10 min. The EC was calculated by the formula:

Electrolyte leakage(%)=[(ECy−ECx)/(ECz)]∗100.

### 2.7 Relative leaf water content

Leaf disks (excluding midrib) from fresh leaves of 2 cm^3^ (55 and 75 days after germination) were weighed (FM) and placed in petri dishes having deionized water for 24 hr (Smart & Bingham, 1974). The clinging water adhering to the disks was wiped, and turgor mass (TM) was measured. The dry mass (DM) was noted by placing the samples in the oven for 24 hr at 80°C. Relative leaf water content (RLWC) was calculated by the following formula:

RLWC(%)=[(FM−DM)/(TM−DM)]∗100.

### 2.8 Photosynthetic measurements

The net photosynthesis rate, intercellular carbon dioxide concentration, stomatal conductance, and transpiration rate were determined by portable photosynthetic system LI‐COR 6400XT (LI‐COR 6400XT, Lincoln, NE, USA). The third leaf from the top was selected for measurements from 10:00 am to 12:00 pm on a sunny day after 40 days of AMF inoculation.

### 2.9 AMF colonization

At the end of the experiment, fine cucumber roots (1 cm in length) were collected from each replication and were first treated with 20% HCl followed by a treatment of 10% KOH as determined by Phillips and Hayman (1970). The roots were then thoroughly washed and stained with trypan blue to be clearly evaluated under a light microscope (Olympus, Japan) having a magnification of 50 μm. The colonization percentage was calculated according to Mcgonigle, Miller, Evans, Fairchild, and Swan (1990) on at least 50 root pieces per treatment. The AMF colonization was derived from the formula:

$$\begin{array}{cc} \text{Root colonization}\;(\%)= & [\text{No of colonized roots pieces}/\\ & \text{No of observed roots pieces}]*100. \end{array}$$

### 2.10 Antioxidant enzymes

Healthy 0.5 g of leaf samples was collected from all of the treatments in replicate and grounded in liquid nitrogen. They were homogenized in phosphate buffer (0.05 mol/L phosphate buffer, pH 7.8) having 1% (w/v) polyvinylpyrrolidone (PVP). The solution was transferred to tubes and was centrifuged at 12,000 g at a temperature of 4°C for 20 min. The supernatant obtained was used for assaying the activities of superoxide dismutase (SOD; EC 1.15.1.1), peroxidase (POD; EC 1.11.1.7), catalase (CAT; EC 1.11.1.6), and malondialdehyde content (MDA) (Gao, 2006).

Total SOD was measured by the inhibition of the photochemical reduction of nitroblue tetrazolium (NBT) generated by superoxide radicals (Stewart & Bewley, 1980). The reaction mixture consisted of 1.5 ml phosphate buffer (0.05 M, pH 7.8), 0.3 ml (0.75 mmol/L) NBT, 0.3 ml (0.02 mmol/L) riboflavin, 0.3 ml (0.1 mmol/L) EDTA‐Na_2_, 0.3 ml (0.13 mol/L) methionine, 0.25 ml distilled water, and 0.05 ml enzymatic extract. This extract was subjected to fluorescent light exposure (86.86 μmol m^−2^ s^−1^) for 15–20 min, and the change in color absorbance was deducted at 560‐nm wavelength on a spectrophotometer (UV‐3802, UNICO, MDN, USA). The total SOD activity was expressed in units per gram of fresh leaves (μg^−1^ Fw hr^−1^).

POD activity was determined through guaiacol method (Polle, Otter, & Seifert, 1994). The reaction mixture includes 0.1 ml 0.05 M phosphate buffer (pH 7.8), 28 microliter guaiacol, 19 μl 30% H_2_O_2_ (v/v), and 0.5 ml enzyme extract. The absorbance was recorded at 470‐nm wavelength at 30‐second intervals for 3 mins. The results are described as D_470_ per minute per gram of fresh leaves (μg g^−1^ FW min^−1^).

CAT activity was assayed by measuring H_2_O_2_ reduction (Chance & Maehly, 1955). The reaction mixture includes 1.9 ml of (200 mmol/L, pH, 7.0) phosphate buffer, 1 ml 30% H_2_O_2_, and 0.1 ml enzyme extract. The H_2_O_2_ reduction was recorded at 240‐nm wavelength after every 30‐s till 3 min. The activity of CAT is presented as OD 240 nm (μg g^−1^ FW min^−1^).

The MDA content was measured through thiobarbituric acid (TBA) reaction method (Dhindsa, Plumb‐Dhindsa, & Reid, 1982). The enzyme extract of 1.5 ml was mixed with 2 ml of 0.6% (w/v) TBA solution dissolved in 5% (v/v) trichloroacetic acid (TCA). The extract was heated in a water bath for 10 min and cooled to room temperature to allow the precipitate to settle down. The supernatant was used for the spectrophotometric determination of MDA at 450‐ and 532‐nm wavelength and subtracted from the absorbance at 600 nm. The MDA content was expressed as the amount of substance per gram of fresh leaves (nmol/g Fw).

### 2.11 Plant nutrients

Cucumber shoot and root samples were oven‐dried and ground, and 0.5 g of samples was digested in boiling hydrochloric acid with addition of hydrogen peroxide till solution becomes clear. After cooling, the volume of the solution was raised to 100 ml with distilled water. Nitrogen was determined in shoot and roots by modified micro‐Kjeldahl method (Steyermark, 1961). Phosphorous was determined through spectrophotometer (Olsen, Cole, Watandbe, & Dean, 1954). Potassium and sodium were determined through flame photometer connected with continuous‐flow systems (microflow automated continuous‐flow analyzer III, Italy).

### 2.12 Statistical analysis

Experimental treatments were arranged in a split plot design with salt stress as main plot and HBL, AMF, and their combination as subplot having three biological replicates. Results were subjected to analysis of variance (ANOVA), and their means were separated according to the least significant difference (LSD) at 0.05 level probability using SPSS statistical program. Sigma plot v.12.5 program was used for the making of graphs.

## 3 RESULTS

### 3.1 Growth attributes

According to our results, the NaCl stress decreased shoot and root length and fresh and dry weight of cucumber plants in both of the cultivars. As shown in Table 1, the HBL spraying intervals (after 15 days and 30 days) alone and in combination with AMF showed increment in plant growth and biomass under NaCl stress. However, the HBL spray after 15 days in combination with AMF showed highest results in the shoot and root length and fresh and dry weight in cultivar Jinyou 1^#^ and CCMC.

**Table 1:** Regulation of shoot length, root length, fresh weight, and dry weight by HBL spraying intervals, AMF, and their combination on cucumber cultivars Jinyou 1^#^ and CCMC under NaCl (100 mmol/L), respectively

Cultivars	Treatments		Shoot length (cm)	Root length (cm)	Shoot fresh weight (g)	Shoot dry weight (g)	Root fresh weight (g)	Root dry weight (g)
Jinyou 1#	NaCl (0 mmol/L)	Control	92.2 ± 2.2 c	54.7 ± 2.5 c	98.3 ± 1.8 c	14.9 ± 1.7 c	18.1 ± 2.3 c	3.3 ± 0.9 c
		HBL 30 days	92.8 ± 2.5 c	52.3 ± 2.4 c	98.7 ± 1.3 c	15.2 ± 0.9 c	18.2 ± 2.5 c	3.3 ± 0.9 bc
		HBL 15 days	95.4 ± 2.1 b	56.8 ± 2.6 ab	101.6 ± 1.6 b	15.8 ± 1.2 bc	18.8 ± 2.2 bc	3.4 ± 0.8 b
		AMF	93.6 ± 2.4 c	55.4 ± 2.4 c	99.6 ± 0.9 c	15.8 ± 0.8 bc	18.8 ± 2.2 bc	3.4 ± 0.5 b
		HBL 30 days + AMF	96.7 ± 1.9 b	57.2 ± 3.6 b	102 ± 1.4 ab	16.3 ± 1.3 b	19.3 ± 2.4 ab	3.7 ± 0.9 a
		HBL 15 days + AMF	97.7 ± 1.8 a	58.2 ± 2.3 a	103.7 ± 1.3 a	17.2 ± 1.1 a	20.1 ± 2.6 a	3.7 ± 1.1 a
	NaCl (100 mmol/L)	Control	65.7 ± 2.3 g	36.7 ± 2.6 g	64.6 ± 0.8 g	7.8 ± 0.2 g	8.1 ± 1.2 g	0.8 ± 0.4 g
		HBL 30 days	73.1 ± 1.6 f	41.6 ± 2.1 f	73.7 ± 1.6 f	10.8 ± 0.4 ef	11.6 ± 1.5 f	1.3 ± 0.7 e
		HBL 15 days	75.6 ± 2.1 e	42.8 ± 2.9 e	74.5 ± 1.4 ef	10.9 ± 0.2 ef	13.2 ± 1.2 de	1.4 ± 0.9 de
		AMF	72.1 ± 2.2 f	41.5 ± 2.3 f	75.2 ± 0.6 de	10.5 ± 0.3 f	12.7 ± 1.1 e	1.3 ± 0.8 f
		HBL 30 days + AMF	76.8 ± 1.7 e	42.9 ± 2.8 de	75.9 ± 0.5 d	11.6 ± 0.7 e	13.3 ± 1.2 de	1.5 ± 0.6 d
		HBL 15 days + AMF	79.7 ± 1.4 d	43.9 ± 2.2 d	76.4 ± 1.4 d	12.8 ± 0.4 d	13.9 ± 0.9 d	1.5 ± 0.9 d
CCMC	NaCl (0 mmol/L)	Control	92.1 ± 2.6 b	51.8 ± 2.4 b	100.4 ± 1.7 c	17.3 ± 1.7 c	16.5 ± 2.4 c	3.6 ± 0.8 c
		HBL 30 days	92.2 ± 1.8 b	52.4 ± 2.6 b	101.6 ± 1.6 c	16.9 ± 1.6 c	16.6 ± 2.2 bc	3.6 ± 0.5 bc
		HBL 15 days	93.3 ± 1.9 b	54.9 ± 3.2 a	102.9 ± 1.4 b	17.7 ± 0.9 bc	16.9 ± 1.9 bc	3.6 ± 0.7 bc
		AMF	93.7 ± 1.7 b	52.9 ± 2.8 b	100.8 ± 2.1 c	17.8 ± 1.2 bc	17 ± 1.6 bc	3.7 ± 1 b
		HBL 30 days + AMF	96.1 ± 1.8 a	55.1 ± 2.3 a	103.1 ± 1.3 b	18.6 ± 1.8 ab	17.4 ± 1.8 b	3.8 ± 0.7 a
		HBL 15 days + AMF	96.7 ± 2.2 a	56.3 ± 2.6 a	104.4 ± 1.4 a	19.2 ± 1.2 a	18.7 ± 1.4 a	3.9 ± 0.6 a
	NaCl (100 mmol/L)	Control	64.2 ± 1.4 f	33.5 ± 1.8 f	66.2 ± 0.8 h	9.8 ± 1 f	6.1 ± 1.1 g	1 ± 0.04 f
		HBL 30 days	71.6 ± 0.9 e	39.5 ± 2.3 e	77.7 ± 0.7 g	13.1 ± 1.2 e	9.4 ± 1.1 f	1.6 ± 0.3 e
		HBL 15 days	75.1 ± 1.3 d	41.1 ± 1.9 d	76.1 ± 0.8 fg	13.3 ± 0.8 e	11.5 ± 1.7 de	1.7 ± 0.6 de
		AMF	71.9 ± 1.4 e	39.3 ± 1.5 e	76.8 ± 0.6 ef	12.8 ± 0.9 e	11.1 ± 1.4 e	1.7 ± 0.7 de
		HBL30 days + AMF	76.5 ± 0.8 d	42.2 ± 1.7 cd	77.7 ± 0.3 de	13.6 ± 1.1 e	11.8 ± 1.6 de	1.7 ± 0.2 de
		HBL 15 days + AMF	79.5 ± 1.1 c	42.6 ± 1.6 c	78.3 ± 0.8 d	15.3 ± 0.5 d	12.2 ± 1.4 d	1.8 ± 0.02 d

Data are means of three replicates ± standard error. Means followed by the same letters in a column are not significantly different at *p* = .05 using LSD.

### 3.2 Chlorophyll and root activity

As presented in Table 2, the NaCl at 100 mmol/L reduced chlorophyll a, b, a+b, and root activity in both cultivars. The HBL spraying intervals (after 15 and 30 days) alone and in combination showed significant results as compared to control plants; however, the HBL spraying interval after 15 days in combination with AMF showed increment in chlorophyll a, a+b, and root activity by 24%, 32%, and 24% in cultivar Jinyou 1^#^ and chlorophyll a, b, a+b, and root activity by 25%, 57%, 30%, and 26% in cultivar CCMC.

**Table 2:** Regulation of chlorophyll a, b, a+b (mg/g FW), and root activity (mg g^−1^ hr^−1^) by HBL spraying intervals, AMF, and their combination on cucumber cultivars Jinyou 1# and CCMC under NaCl (100 mmol/L), respectively

Cultivars	Treatments		Chlorophyll a (mg/g FW)	Chlorophyll b (mg/g FW)	Chlorophyll a+b (mg/g FW)	Root activity (mg g^−1 ^hr^−1^)
Jinyou 1#	NaCl (0 mmol/L)	Control	13.5 ± 1.3 b	2.9 ± 0.9 d	16.5 ± 1.5 c	21.9 ± 2.3 d
		HBL 30 days	13.9 ± 2.1 ab	3 ± 0.6 d	16.9 ± 1.3 bc	21.9 ± 3.1 d
		HBL 15 days	14.1 ± 1.9 ab	3.3 ± 0.8 bc	17.3 ± 1.8 ab	22.1 ± 3.2 cd
		AMF	14.2 ± 1.7 ab	3.2 ± 0.5 c	17.4 ± 1.2 ab	22.4 ± 2.6 bc
		HBL 30 days + AMF	14.4 ± 1.4 a	3.4 ± 0.5 ab	17.8 ± 1.6 a	22.6 ± 2.4 ab
		HBL 15 days + AMF	14.5 ± 1.7 a	3.5 ± 0.5 a	17.9 ± 1.4 a	22.9 ± 2.7 a
	NaCl (100 mmol/L)	Control	9.2 ± 0.4 e	0.7 ± 0.2 g	9.9 ± 1.2 g	10.5 ± 2.1 h
		HBL 30 days	10.5 ± 0.7 d	1.3 ± 0.2 f	11.8 ± 1.1 f	12.6 ± 2.5 g
		HBL 15 days	10.9 ± 1.1 cd	1.4 ± 0.3 f	12.3 ± 0.9 ef	12.6 ± 2.2 g
		AMF	10.2 ± 0.4 d	1.3 ± 0.1 f	11.6 ± 1.3 f	12.7 ± 2.2 fg
		HBL 30 days + AMF	11.3 ± 0.8 c	1.6 ± 0.3 e	12.8 ± 1.2 de	12.9 ± 2.3 ef
		HBL 15 days + AMF	11.5 ± 1 c	1.5 ± 0.4 e	13.1 ± 1.1 d	13.2 ± 2.5 e
CCMC	NaCl (0 mmol/L)	Control	16.1 ± 2.2 b	3.8 ± 0.2 d	19.8 ± 1.3 d	22.7 ± 2.7 c
		HBL 30 days	16 ± 1.7 ab	3.9 ± 0.3 d	19.9 ± 1.4 cd	22.8 ± 2.7 c
		HBL 15 days	16.3 ± 2.3 ab	4.1 ± 0.6 bc	20.4 ± 1.2 abc	23.1 ± 2.1 bc
		AMF	16.2 ± 1.5 ab	4.1 ± 0.4 c	20.3 ± 0.9 bcd	23.2 ± 2.9 bc
		HBL 30 days + AMF	16.4 ± 1.3 ab	4.2 ± 0.2 ab	20.6 ± 1.8 ab	23.5 ± 2.5 ab
		HBL 15 days + AMF	16.5 ± 0.9 a	4.3 ± 0.5 a	20.8 ± 1.5 a	23.8 ± 2.8 a
	NaCl (100 mmol/L)	Control	10.8 ± 0.5 f	1.5 ± 0.2 g	12.4 ± 0.8 h	11.4 ± 2.1 f
		HBL 30 days	11.9 ± 0.6 e	2.1 ± 0.4 f	14 ± 1.2 g	13.4 ± 2.3 e
		HBL 15 days	12.7 ± 0.7 d	2.2 ± 0.2 f	14.9 ± 0.7 f	13.5 ± 2.3 e
		AMF	11.9 ± 0.9 e	2.2 ± 0.3 f	14.1 ± 1.1 g	13.6 ± 2.8 e
		HBL30 days + AMF	13.4 ± 1.1 c	2.4 ± 0.5 e	15.7 ± 1.4 e	13.8 ± 2.3 e
		HBL 15 days + AMF	13.6 ± 1.3 c	2.5 ± 0.4 e	16.1 ± 1.5 e	14.4 ± 2.2 d

Data are means of three replicates ± standard error. Means followed by the same letters in a column are not significantly different at *p* = .05 using LSD.

### 3.3 EC and RLWC

**Figure 1:**
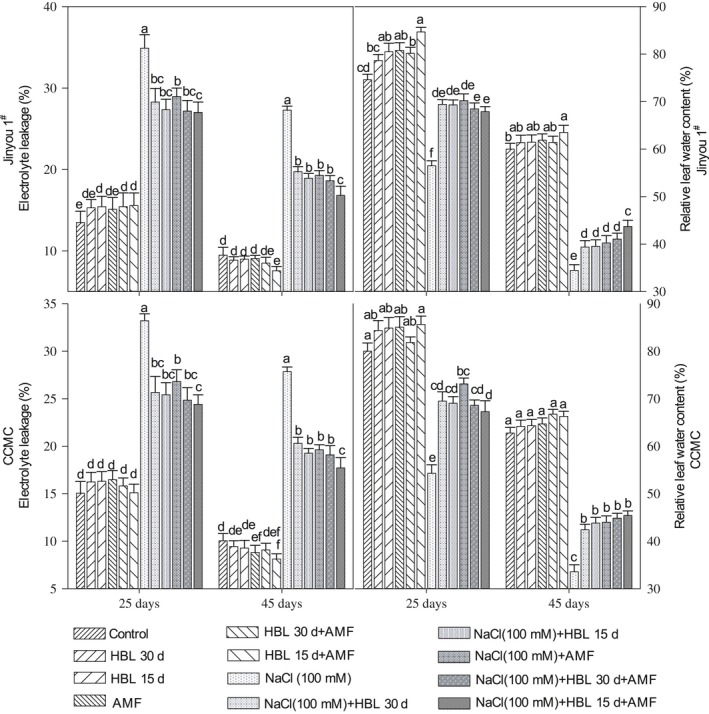
Effect of HBL spraying intervals, AMF, and their combination on electrolyte leakage and relative leaf water content of leaves after 25 and 45 days in cucumber cultivars Jinyou 1# and CCMC under NaCl (100 mmol/L), respectively. Data are means of three replicates* *±* *standard error. Means followed by the same letters in a column are not significantly different at p = .05 using LSD

Salinity stress increased EC after 25 and 45 days of salt treatments by 150% and 180% in cultivar Jinyou 1^#^ and 120% and 170% in cultivar CCMC, respectively. As depicted in Figure 1, the HBL spraying intervals, AMF, and their combination significantly reduced EC after 25 and 45 days of salt treatments. However, the HBL spraying interval after 15 days in combination with AMF decreased EC by 22% and 38% in cultivar Jinyou 1^#^ and 26% and 36% in cultivar CCMC.

Relative leaf water content was significantly decreased in plants under salt stress by 24% and 42% in cultivar Jinyou 1^#^ and 32% and 46% in cultivar CCMC as shown in Figure 1. The HBL spraying intervals, AMF, and their combination significantly increased RLWC in leaves. The highest values were obtained in the HBL spraying interval after 15 days in combination with AMF, which increased RLWC by 20% and 26% in cultivar Jinyou 1^#^ and 23% and 35% in cultivar CCMC.

### 3.4 Photosynthetic parameters

**Figure 2:**
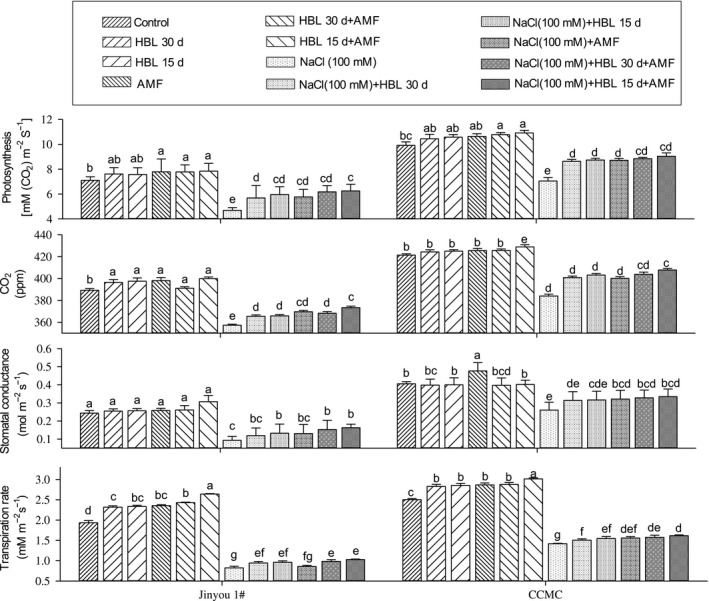
Effect of HBL spraying intervals, AMF, and their combination on photosynthesis [mmol/L(co_2_)m^−2^ s^−1^], carbon dioxide (ppm), stomatal conductance (mol m^−2^ s^−1^), and transpiration rate (mmol/L m^−2^ s^−1^) on cucumber cultivars Jinyou 1^#^ and CCMC under NaCl (100 mmol/L), respectively. Data are means of three replicates* *±* *standard error. Means followed by the same letters in a column are not significantly different at p = .05 using LSD

Figure 2 shows that salinity significantly reduced net photosynthetic rate, carbon dioxide exchange rate, stomatal conductance, and transpiration rate by 33%, 85%, 51%, and 48% in cultivar Jinyou 1^#^ while 32%, 9%, 50%, and 45% in cultivar CCMC as compared to their respective controls. The HBL spraying intervals and in combination with AMF showed increment in the above parameters; however, the highest values were observed in the combined effect of HBL spraying interval after 15 days with AMF, which increased photosynthesis, carbon dioxide exchange rate, stomatal conductance, and transpiration rate under NaCl stress by 33%, 4%, 54%, and 24% in cultivar Jinyou 1^#^ and 39%, 6%, 57%, and 28% in cultivar CCMC.

### 3.5 Colonization percentage

**Figure 3:**
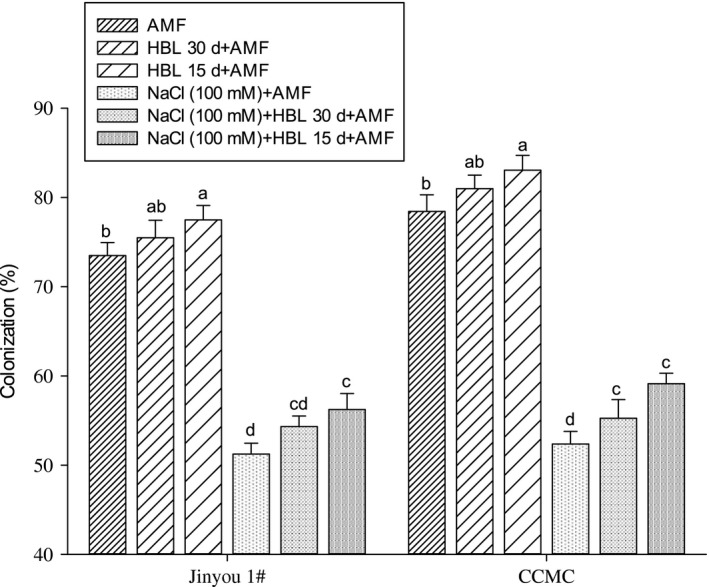
Effect of HBL spraying intervals, AMF, and their combination on AMF colonization in roots of cucumber cultivars Jinyou 1^#^ and CCMC under NaCl (100 mmol/L), respectively. Means followed by the same letters in a column are not significantly different at p = .05 using LSD

As compared to control plants, the colonization percentage was significantly reduced in plants with NaCl (100 mmol/L) stress by 32% in cultivar Jinyou 1^#^ and 30% in cultivar CCMC, respectively (Figure 3). The HBL spraying intervals and in combination with AMF increased colonization percentage in cucumber roots; however, the prominent results were noted in plants treated with the combination of AMF and HBL spraying interval after 15 days. The colonization percentage was increased by 9% in cultivar Jinyou 1^#^ and 12% in cultivar CCMC as compared to their respective controls.

### 3.6 Nitrogen, phosphorous, potassium, and sodium

Our results show that (Table 3 **)** NaCl (100 mmol/L) stress significantly reduced nitrogen and phosphorus in both shoots and roots of cultivar Jinyou 1^#^ and CCMC, respectively. Potassium showed the same trend in shoots of both cultivars; however, on the one hand, its concentration showed increment in roots. On the other hand, sodium concentration was significantly increased in both shoots and roots of both cultivars. The HBL spraying interval and in combination with AMF showed ameliorative role in stress by decreasing sodium ion concentrations; the prominent results, however, were observed in plant treated with HBL spraying interval after 15 days in combination with AMF in both cultivars.

**Table 3:** Regulation of shoot and root nitrogen, phosphorus, potassium, and sodium (μg/L) by HBL spraying intervals, AMF, and their combination on cucumber cultivars Jinyou 1# and CCMC under NaCl (100 mmol/L), respectively

Cultivar	Treatment		Nitrogen (μg/L)		Phosphorus (μg/L)		Potassium (μg/L)		Sodium (μg/L)		K/Na ratio	
			Shoot	Root	Shoot	Root	Shoot	Root	Shoot	Root	Shoot	Root
Jinyou 1#	NaCl (0 mmol/L)	Control	46.3 ± 2.6 c	13.7 ± 2.3 d	43.3 ± 3.2 c	104.5 ± 3.1 d	83.2 ± 2.8 c	86.9 ± 3.5 d	23.9 ± 1.2 f	29.1 ± 0.5 f	3.5 ± 0.2 e	2.9 ± 0.3 d
		HBL 30 days	46.5 ± 2.8 c	13.9 ± 1.6 cd	43.6 ± 3.1 c	105.1 ± 2.6 cd	84.4 ± 1.9 c	87.6 ± 2.8 d	22.7 ± 1.4 f	26.4 ± 0.6 g	3.7 ± 0.2 d	3.3 ± 0.4 c
		HBL 15 days	48.4 ± 1.9 b	14.7 ± 1.7 bc	43.8 ± 2.7 c	105.3 ± 1.7 c	85.1 ± 1.5 bc	88.7 ± 1.6 d	21.4 ± 1.1 g	26.1 ± 0.5 gh	3.9 ± 0.3 c	3.4 ± 0.2 bc
		AMF	48.6 ± 1.5 b	14.9 ± 1.9 bc	45.3 ± 2.5 b	106.9 ± 2.1 b	86.1 ± 2.6 b	89.9 ± 1.6 cd	19.2 ± 0.5 h	25.9 ± 0.4 gh	4.5 ± 0.1 b	3.5 ± 0.1 b
		HBL 30 days + AMF	48.9 ± 2 ab	15.5 ± 0.9 ab	45.6 ± 2.6 ab	108.9 ± 1.8 a	90.2 ± 2.3 a	83.5 ± 2.1 e	18.8 ± 0.4 h	25.2 ± 0.4 hi	4.8 ± 0.2 a	2.5 ± 0.3 e
		HBL 15 days + AMF	49.7 ± 1.3 a	16.2 ± 1.2 a	46.9 ± 2.1 a	109.5 ± 1.3 a	91.1 ± 1.7 a	92.1 ± 1.4 c	18.5 ± 0.2 h	24.5 ± 0.7 i	4.9 ± 0.1 a	3.8 ± 0.5 a
	NaCl (100 mmol/L)	Control	30.6 ± 2.4 g	8 ± 0.7 g	28.2 ± 1.8 g	66.9 ± 1.6 h	49.9 ± 1.6 h	138.7 ± 3.5 a	156.7 ± 3 a	149 ± 3.6 a	0.3 ± 0.01 g	0.8 ± 0.01 g
		HBL 30 days	41.3 ± 0.9 f	8.6 ± 0.7 fg	33.9 ± 2.8 f	78.9 ± 2.6 g	57.5 ± 1.2 g	123.6 ± 2.9 ab	94.4 ± 2.4 b	124.1 ± 3.5 b	0.6 ± 0.01 f	1.1 ± 0.1 f
		HBL 15 days	41.8 ± 1.6 f	9.6 ± 1.2 e	34.3 ± 1.7 f	79.3 ± 1.4 g	58.1 ± 0.9 g	121.7 ± 2.4 b	90.7 ± 2.9 c	123.2 ± 3.1 b	0.6 ± 0.02 f	1.1 ± 0.1 f
		AMF	42.9 ± 1.5 e	8.9 ± 0.6 ef	35.3 ± 1.5 f	80.5 ± 1.7 f	57.9 ± 0.8 g	121.2 ± 1.7 b	89.3 ± 2.7 d	121.9 ± 2.7 c	0.7 ± 0.02 f	1.2 ± 0.1 f
		HBL 30 days + AMF	43.5 ± 1 de	9.2 ± 0.5 ef	36.34 ± 2.1 d	83.4 ± 2.4 e	60.7 ± 1.4 f	117.4 ± 2.1 b	88.2 ± 1.7 d	119.4 ± 2.6 d	0.7 ± 0.01 f	1.2 ± 0.2 f
		HBL 15 days + AMF	44.2 ± 1.6 d	9.6 ± 1.1 e	36.9 ± 1.8 d	83.7 ± 1.9 e	62.7 ± 1.3 e	113.9 ± 1.3 bc	85.5 ± 1.9 e	118.1 ± 2.4 e	0.8 ± 0.02 f	1.3 ± 0.2 f
CCMC	NaCl (0 mmol/L)	Control	51.8 ± 1.6 b	16.5 ± 1.5 c	44.4 ± 2.8 b	107.6 ± 3.5 c	84.8 ± 2.9 cd	85.1 ± 2.6 d	27.6 ± 1.7 f	26.3 ± 0.5 f	3.1 ± 0.2 c	3.2 ± 0.3 d
		HBL 30 days	52.4 ± 2.6 b	16.8 ± 0.8 c	49.2 ± 1.6 b	110.7 ± 2.3 b	85.6 ± 2.4 c	85.8 ± 2.1 d	25.9 ± 1.3 g	23.7 ± 1.2 g	3.3 ± 0.3 b	3.6 ± 0.2 c
		HBL 15 days	52.6 ± 1.8 b	17.9 ± 1.9 b	49.8 ± 2.8 b	110.8 ± 1.8 b	85.9 ± 2.8 c	86.9 ± 3.4 d	25.7 ± 1.6 g	23.4 ± 1.3 gh	3.3 ± 0.2 b	3.7 ± 0.5 bc
		AMF	52.9 ± 2.4 ab	17.8 ± 1.2 b	50.1 ± 2.2 ab	110.9 ± 2 b	86.9 ± 2.5 b	88.1 ± 1.7 cd	25.5 ± 0.9 g	23.1 ± 0.8 gh	3.4 ± 0.3 b	3.8 ± 0.4 b
		HBL 30 days + AMF	53.9 ± 2.2 a	18.3 ± 0.6 b	51.6 ± 1.8 a	112.7 ± 2.2 a	92.9 ± 2.3 a	84.7 ± 3.6 e	24.7 ± 1 gh	22.5 ± 1.5 hi	3.8 ± 0.2 a	2.8 ± 0.2 e
		HBL 15 days + AMF	54.1 ± 1.2 a	19.2 ± 1.4 a	53.3 ± 1.4 a	112.9 ± 1.8 a	93.3 ± 2.6 a	90.2 ± 1.6 c	24.2 ± 0.7 h	21.8 ± 0.5 i	3.9 ± 0.3 a	4.1 ± 0.1 a
	NaCl (100 mmol/L)	Control	34.9 ± 2.5 f	11.2 ± 0.3 f	34.7 ± 1.1 f	70.1 ± 1.6 f	51.8 ± 1.5 h	136.9 ± 3.4 a	143.4 ± 1 a	146.3 ± 3.5 a	0.4 ± 0.01 e	0.7 ± 0.01 g
		HBL 30 days	45.4 ± 1.7 e	11.6 ± 1.1 ef	40.2 ± 0.8 de	82.4 ± 1.4 e	58.9 ± 1.2 g	121.8 ± 2.6 ab	91.3 ± 0.8 b	121.4 ± 3.7 b	0.6 ± 0.02 d	0.9 ± 0.1 f
		HBL 15 days	45.8 ± 2.1 de	12.5 ± 0.7 d	40.6 ± 0.7 d	82.6 ± 1.8 e	59.6 ± 0.7 g	119.9 ± 1.8 ab	90.3 ± 0.3 b	120.4 ± 2.8 b	0.7 ± 0.03 d	1 ± 0.03 f
		AMF	46.2 ± 1.6 de	11.9 ± 1 def	41.4 ± 0.9 cd	82.8 ± 1.5 e	59.7 ± 0.6 fg	119.4 ± 1.4 b	88.5 ± 0.3 c	119.2 ± 3.3 c	0.7 ± 0.01 d	1.1 ± 0.2 f
		HBL 30 days + AMF	46.8 ± 2.2 cd	12.2 ± 0.4 de	42.6 ± 0.4 cd	86.3 ± 1.1 d	61.8 ± 0.3 f	115.6 ± 1.2 b	86.7 ± 0.6 d	116.6 ± 2.2 d	0.7 ± 0.03 d	1.1 ± 0.2 f
		HBL 15 days + AMF	47.8 ± 1.3 c	12.6 ± 0.5 d	42.9 ± 1.6 c	86.7 ± 1.4 d	63.8 ± 0.6 e	112.1 ± 1.3 b	84.9 ± 0.5 e	115.4 ± 2.5 e	0.8 ± 0.02 d	1.2 ± 0.3 f

Data are means of three replicates ± standard error. Means followed by the same letters in a column are not significantly different at *p* = .05 using LSD.

### 3.7 Plant antioxidant enzymes

#### 3.7.1 Superoxide dismutase

**Figure 4:**
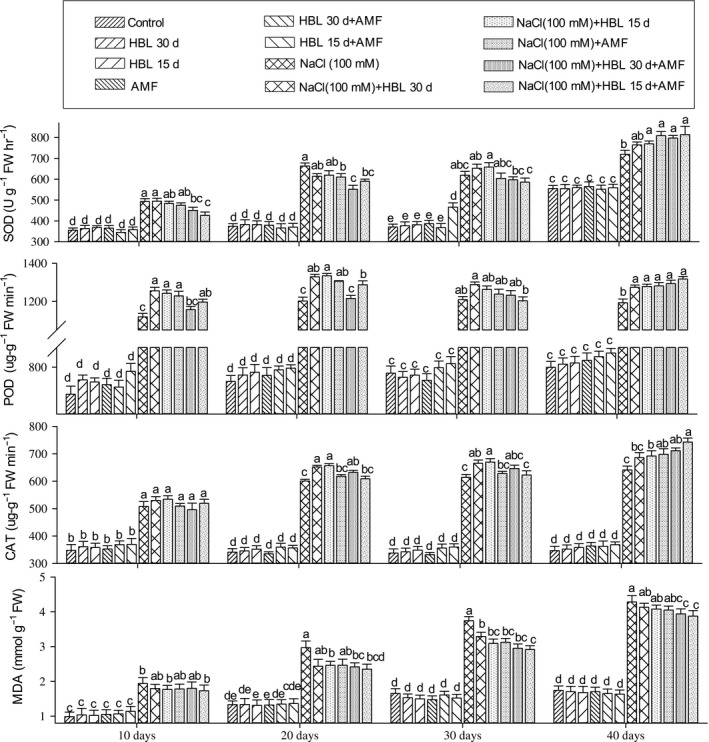
Effect of HBL spraying intervals, AMF, and their combination on antioxidant enzymes, superoxide dismutase (U g^−1^ FW hr^−1^), peroxidase (μg g^−1^ FW min^−1^), catalase (μg g^−1^ FW min^−1^), and malondialdehyde content (mmol/g FW) of cucumber cultivar Jinyou 1^#^ under NaCl (100 mmol/L). Data (means ± SD, n = 3) sharing the same letters above the bars are not significantly different at p = .05

**Figure 5:**
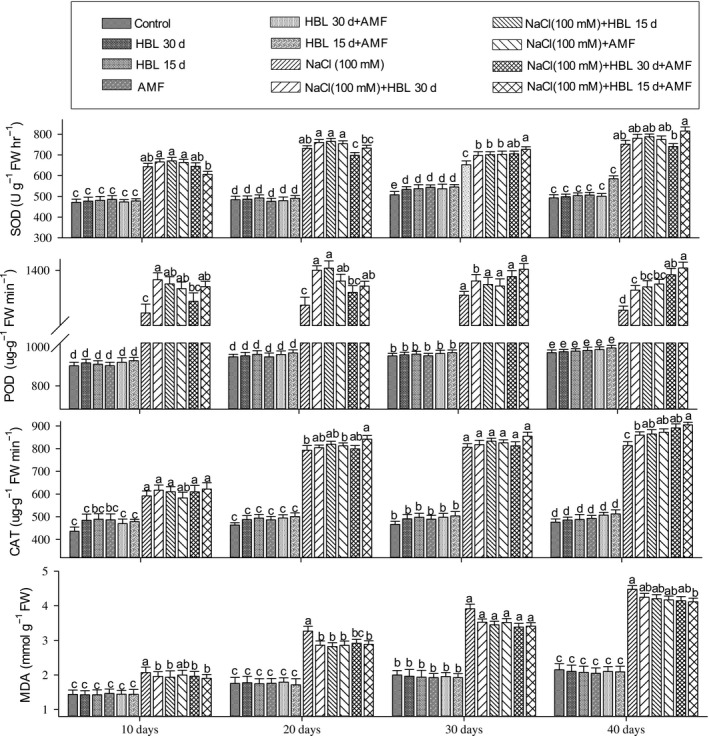
Effect of HBL spraying intervals, AMF, and their combination on antioxidant enzymes, superoxide dismutase (U g^−1^ FW hr^−1^), peroxidase (μg g^−1^ FW min^−1^), catalase (μg g^−1 ^FW min^−1^), and malondialdehyde content (mmol/g FW) of cucumber cultivar CCMC under NaCl (100 mmol/L). Data (means ± SD, n = 3) sharing the same letters above the bars are not significantly different at p = .05

In our results, the SOD activity in plants under NaCl (100 mmol/L) stress increased after 10 days; however, it decreased after 20 days in cultivar Jinyou 1^#^ (Figure 4) and 30 days in cultivar CCMC (Figure 5), respectively. The HBL foliar spray interval after 15 and 30 days reduced stress conditions by increasing SOD activity after every 10 days. The AMF alone showed a slow increment after every 10 days, but the prominent results were observed in HBL spraying interval after 15 days in combination with AMF. The SOD activity showed increment after every 10 days, and it showed the highest results after 40 days of NaCl treatments.

#### 3.7.2 Peroxidase

According to Figures 4 and 5, in the plants under NaCl (100 mmol/L), POD activity increased after 10 days but declined after 20 days in cultivar Jinyou 1^#^ (Figure 4) and CCMC (Figure 5), respectively. The HBL foliar spray interval after 15 and 30 days showed increment in POD activity after every 10 days. The same trend was observed in AMF treatments of both cultivars, but the highest values were obtained in HBL spraying interval after 15 days in combination with AMF where the POD activity was the highest after 40 days of NaCl (100 mmol/L).

#### 3.7.3 Catalase

As compared to control plants, CAT activity showed gradual increase after every 10 days under NaCl (100 mmol/L) stress in cultivars Jinyou 1^#^ (Figure 4) and CCMC (Figure 5), respectively. The HBL spraying interval after 15 and 30 days and AMF treatments showed the same trend of gradual increase in both cultivars; however, the maximum results were noted in plants treated with HBL spraying interval of 15 days in combination with AMF under salt stress.

#### 3.7.4 Malondialdehyde

As shown in Figures 4 and 5, the MDA content in the plants under NaCl (100 mmol/L) stress increased after every 10 days in cultivars Jinyou 1^#^ and CCMC, respectively. The HBL spraying interval after 15 and 30 days and AMF treatment gradually decreased MDA content after every 10 days; however, the significant results were observed in HBL spraying interval after 15 days in combination with AMF. The MDA content declined after 10, 20, 30, and 40 days of both cultivars under salt stress.

## 4 DISCUSSION

Salinity is a major obstacle in the modern agriculture industry and is the main reason of declining valuable produce in this era (Qadir et al., 2014). Some strategies such as plant growth regulators or AMF have been documented to be involved in increasing growth and production under stress conditions (Foo et al., 2013; Ruiz‐Lozano et al., 2012). Our research findings are novel to consider the combined effect of AMF and HBL spraying intervals in alleviating salinity in cucumber. The findings provide an insight into the combined effects of HBL spraying intervals and AMF (abiotic and biotic interaction) to have improved the plant biochemical responses in saline conditions. It has been reported that AMF colonization in host roots is reduced under stress conditions, thus affecting growth and physiology of plants (Colebrook, Thomas, Phillips, & Hedden, 2014; Foo, Ferguson, & Reid, 2014; Miransari, Abrishamchi, Khoshbakht, & Niknam, 2014); our results revealed that the foliar application of HBL spraying intervals after 15 days in combination with AMF reduced stress effects in both cultivars. However, the salt‐tolerant cultivar CCMC revealed improved growth and biomass as compared to sensitive cultivar Jinyou 1^#^. According to Hajiboland et al. (2010), the efficient symbiosis was established with salt‐tolerant cultivar as their roots were less affected by salt stress as compared to salt‐sensitive cultivars, thus resulting in more protection and improved growth. Studies have shown that foliar application of HBL has an ameliorative role in reducing stress conditions by increasing shoot and root length in lettuce (Ekinci, Yildirim, Dursun, & Turan, 2012), cucumber (Ahmad et al., 2017), and wheat (Hayat et al., 2014). The improved growth and biomass of cultivar CCMC under the combination of HBL spraying intervals and AMF suggest its tolerance nature, efficient water, and nutrient absorption as compared to sensitive cultivar under stress conditions. Similarly, the improved growth attributes were also reported in plants where AMF was in combination with salicylic acid (Ghazanfar et al., 2015), endophytic bacteria (Hashem et al., 2016), and gibberellic acid (Khalloufi et al., 2017).

In our results, the chlorophyll content, photosynthesis, and root activity were decreased in both cultivars, while the cultivar CCMC exhibited a slight tolerance as compared to cultivar Jinyou 1^#^ under salt stress. The increased activity of chlorophyllase under salt stress damages chlorophyll content and decreases photosynthetic activity, stomatal conductance, and modify source/sink relationship (Hashem et al., 2015; Sarwat et al., 2016). Stomata are the main structures responsible for leaf gaseous exchange, salinity restricts the availability of atmospheric CO_2_ due to stomatal closure, and consequently, the consumption of NADPH is reduced in Calvin cycle. The over‐reduction in ferredoxin in photosystem I leads to the production of oxygen radicals, which causes a chain reaction of singlet oxygen species (ROS) in a process called Mehler reaction and leads to oxidative stress (Greco, Chiappetta, Bruno, & Bitonti, 2012; Hanin et al., 2016; Mittler, 2002). It is reported that AMF symbiosis causes a fundamental change in physiology and biochemistry of the leaf and hydraulic properties of roots, which results in elevated photosynthesis, transpiration rate, and gaseous exchange (Hajiboland et al., 2010; Pedranzani et al., 2016; Porcel et al., 2012). The increased chlorophyll content, root activity, and photosynthesis in cultivar CCMC treated with HBL spraying intervals after 15 days in combination with AMF can be attributed to the enhanced nutrients and water absorption, increased roots hydraulic activities, and lower uptake of sodium ion. As documented by Hashem et al. (2015) the increase in chlorophyll contents and root activity of plants under AMF might be due to a decrease in concentration of leaves Na^+^ concentration and increase uptake of nutrients, especially Mg^+2^. Reports have also shown that HBL foliar application enhances chlorophyll content and root development, which leads to improved photosynthesis (Fariduddin et al., 2014; Hayat et al., 2014; Niu et al., 2016). The HBL detoxifies salinity effects by enhancing the activity of aquaporins through turgor‐driven cell expansion or by proton pumping in modulating tolerance (Ekinci et al., 2012), which improves nutrients absorption and facilitates photosynthates transfer (Ali et al., 2008) from leaves to sink and improve the overall metabolic activity (Sasse, 2003) of plants.

The ROS produced by oxidative stress and Na^+^ ion accumulation under salt stress damages plant cells, organelles, and proteins (Porcel et al., 2012; Schweiger et al., 2014), while the stomatal closure and poor uptake of water from roots additionally result in the high amount of electrolytes leakage, and decline of relative water content. In our results, the cultivar Jinyou 1^#^ exhibited less tolerance to EC and relative water content of leaves. The increase in leave relative water content and decreased EC in cultivar CCMC under HBL spraying interval after 15 days in combination with AMF can be attributed to the improved water absorption from roots as AMF, on the one hand, have been reported to enhance root hydrolytic activity, cause osmotic balance, and improve nutrient absorption and composition of carbohydrates (Evelin, Kapoor, & Giri, 2009). HBL, on the other hand, regulates cell division, differentiation, and elongation along with enhanced proton pump efficiency to overcome toxic effects caused by Na^+^ ion (Ali et al., 2008). The higher RLWC and lower EC from leaves might be due to the increased uptake of water, which detoxifies Na^+^ ion concentration and results in higher photosynthetic activity and enhanced water absorption (Porcel et al., 2012). Under salt stress, sodium causes specific ion toxicity, and thus, the maintaining of lower Na^+^ levels in plants is a key factor of plant adaptation to salt stress (Kong, Luo, Dong, Eneji, & Li, 2016). In our results, as compared to cultivar Jinyou 1^#^, the cultivar CCMC resulted in higher concentrations of nitrogen, phosphorous, and potassium in both roots and shoots under HBL spraying interval after 15 days in combination with AMF under salt stress. Generally, under stress conditions sodium ion competes with potassium ion, leading to decrease in K^+^ levels in shoots which causes a disturbance in ion hemostasis (Garcia & Zimmermann, 2014) and leads to programmed cell death (Shabala, Bose, Fuglsang, & Pottosin, 2016). The excessive accumulation of sodium ion attributes to disruption of essential cellular metabolism, enzyme activities, protein synthesis, etc. In contrast, potassium plays an important role in plant metabolism, ion hemostasis, opening and closing of stomata, enzymes activity, etc. (Garg & Pandey, 2014). Nitrogen and phosphorus uptake and utilization are hindered under salt stress. Salt disturbs nitrogen metabolism by influencing NO_3_^−^ uptake and utilization in protein synthesis (Talaat & Shawky, 2013), which is the result of ion imbalance in cells especially K^+^/Na^+^ ratio. The K^+^/Na^+^ ratio can be used as a physiological indicator for salt tolerance (Garg & Pandey, 2014). A lower K^+^/Na^+^ ratio might lead to low turgor pressure, ion toxicity, and malfunctioning of proteins necessary for growth (Garg & Pandey, 2014; Porcel et al., 2012). The higher K^+^/Na^+^ ratios in halophytes are one of the prominent features, which indicates greater tolerance by limiting the excessive uptake of sodium ion and distribution within the plant (Shabala et al., 2016). The increased nutrient concentration in cultivar CCMC suggests that AMF reduces salt stress by efficiently absorbing water through its hyphae and efficient nutrient absorption, leading to higher K^+^/Na^+^ ion ratio (Hajiboland et al., 2010; Hashem et al., 2016). Phosphorus is highly immobile in soil, and reports have shown that AMF have a high affinity for P uptake through its hyphae where plants are unable to reach (Bücking & Ambilwade, 2012). The effective N uptake in AMF plants might be due to the enhanced P nutrition (Reynolds, Hartley, Vogelsang, Bever, & Schultz, 2005). A recent study by Hammer, Pallon, Wallander, and Olsson (2011) stated that AMF can selectively uptake K and Ca^+2^ ions, as osmotic equivalents, and avoid sodium uptake. The sodium ion might be kept in root cell vesicles and intraradical fungal hyphae to avoid translocation to shoots. AMF plants also efficiently absorb micronutrients, magnesium, and calcium including iron, zinc, and boron (Garg & Pandey, 2014), which are essential in various plant functions. Similarly, it has also been reported that HBL increases K^+^/Na^+^ ratio and stimulates uptake of N, P, K, and other mineral elements (Ekinci et al., 2012) through enhanced proton pumping of sodium ion, which might be one of the mechanisms in stress tolerance of cucumber cultivars (Bajguz & Hayat, 2009; Hayat et al., 2014). It is interesting to observe the combination of HBL spraying intervals and AMF showed more pronounced effects in improvement of growth parameters, perhaps due to their additive effects as reported by Ghazanfar et al. (2015) and Khalloufi et al. (2017).

**Figure 6:**
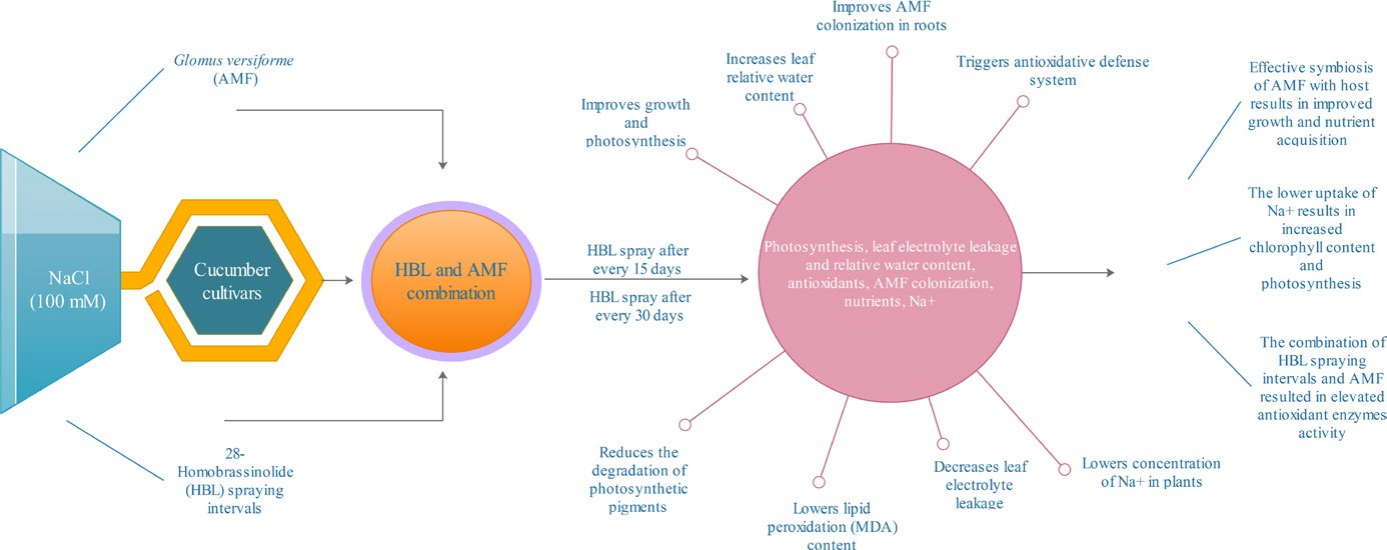
The combination of 28‐homobrassinolide spraying intervals and AMF‐elevated photosynthesis, relative water content, and antioxidant system in leaves as well as AMF colonization in roots. Moreover, it decreased leaf electrolyte leakage and sodium ion concentration in shoots and roots, which resulted in improved growth of cucumber plants under NaCl

It is understood that salt stress produces ROS in plant, which results in oxidative stress under salt stress (Ahmad et al., 1979; Rajewska, Talarek, & Bajguz, 2016). These ROS (consists of superoxide anion, hydroxyl radical, and hydrogen peroxide) are scavenged by plant antioxidant enzymes resulting in reducing the damage to membranes, proteins etc. and even death of the cell. In our results, the decrease in lipid peroxidation (MDA content) and increase in antioxidant enzymes (SOD, POD, and CAT) in cultivar CCMC as compared to cultivar Jinyou 1^#^ under combined effect of HBL spraying interval after 15 days and AMF show the additive effect on plants by causing less damage to membranes. It might also be due to the triggering of antioxidant enzymes, which scavenge the ROS produced and hence lowering the damage caused to membranes (Hayat et al., 2014; Porcel et al., 2012; Rajewska et al., 2016). In our results, the antioxidant activities in cultivar CCMC were higher as compared to cultivar Jinyou 1^#^, which were in accordance with Ahmad et al. (2017), Ali et al. (2008), Hashem et al. (2015), Hayat et al. (2014), and Sarwat et al. (2016) who reported that the inoculation of AMF and HBL as a foliar spray delimits lipid peroxidation under stress conditions. The oxidative stress caused by salt stress activates antioxidant enzyme system (SOD, POD, CAT, PPO etc.), which scavenges ROS, and the balance between SOD, POD, and CAT is crucial for the stability of ROS production (Ahmad et al., 1979). The SOD is the first line of defense and has an affinity for superoxide radical to convert it to H_2_O_2_ and H_2_O under stress conditions. The POD and CAT have different levels of affinity for H_2_O_2_ for converting it to water and oxygen (Mittler, 2002). In our results, the HBL spraying interval after 15 days in combination with AMF‐elevated antioxidants and decreased MDA content after every 10 days in both cultivars. These results can be in relation to Ruiz‐Lozano, Collados, Barea, and Azcón (2001) who stated that AMF colonization under salt stress triggers the antioxidant activity of plants by possessing several SOD genes. The elevated activity of POD and CAT in plants under AMF colonization is correlated with decreased lipid peroxidation levels, which are documented by Hajiboland et al. (2010), Pedranzani et al. (2016), and Sarwat et al. (2016). Reports also show that HBL expressed peroxidase‐encoding genes in Arabidopsis (Goda, 2002), modifies antioxidant enzymes system, and lowers membrane degradation (Ali, Hayat, & Ahmad, 2007; Ali et al., 2008; Cao et al., 2005; Fariduddin et al., 2014; Hayat, Hasan, Yusuf, Hayat, & Ahmad, 2010; Niu et al., 2016) under oxidative, salt, heavy metal, and temperature stress. In our results, the elevated enzymatic activity in the combined effect of HBL spraying interval after 15 days and AMF in both cultivars might be due to the additive effects of HBL and AMF, which is also reported in AMF in combination with salicylic acid (Ghazanfar et al., 2015), endophytic bacteria (Hashem et al., 2016), and gibberellic acid (Khalloufi et al., 2017). The overall findings of our study are illustrated graphically in Figure 6.

## 5 CONCLUSION

28‐Homobrassinolide is a plant hormone, and its application has shown prominent results in several horticultural crops. Based on recent researches, its foliar application can protect plants from various diseases and can increase the quality and quantity of crops. With advancements in genetic modification and production of more stable synthetic equivalents, using BRs could be a practical strategy for improving quality of crops. Although as an emerging chemical, its economic value may be considered somewhat expensive (100 g of HBL costs 300 RMB in P.R. China), yet its utility in very low concentrations can increase crop production potential. In our experiment, only less than a hundred milligrams was used and that makes its cost quite economical. Moreover, it is environment‐friendly and based on current findings, if combined with AMF, the gap between producer needs of growth and consumer health concerns can be decreased considerably. Therefore, to get use of saline soil conditions, using BRs could be a better solution for the commercial production units to obtain sustainable crop growth. Based on these observations may also suggests that HBL and AMF have an additive effect on cucumber cultivars under salt stress. Current findings of our research work on the spraying of HBL in intervals in combination with AMF resulted in improved growth, photosynthesis, efficient nutrient absorption, and elevated antioxidant activities (SOD, POD, and CAT) after every 10 days suggests that HBL and AMF have an additive effect on cucumber cultivars under salt stress. Nonetheless, current findings may be considered to formulate a combination based on HBL spraying interval of 15 days + AMF to improve vegetable production, particularly cucumber under saline conditions as on commercial basis.

## CONFLICT OF INTEREST

None declared.

## AUTHORS’ CONTRIBUTION

Author Husain Ahmad conducted the experiment, recorded the data, and wrote the manuscript. Author Sikandar Hayat helped in recording the data and production of graphs. Author Muhammad Ali and Tao Liu helped in recording the data and analysis of the data. Author Zhihui Cheng supervised, designed, and funded this experiment.
